# Evaluation of the impact of the GRACE risk score on the management and outcome of patients hospitalised with non-ST elevation acute coronary syndrome in the UK: protocol of the UKGRIS cluster-randomised registry-based trial

**DOI:** 10.1136/bmjopen-2019-032165

**Published:** 2019-09-05

**Authors:** Colin C Everett, Keith AA Fox, Catherine Reynolds, Catherine Fernandez, Linda Sharples, Deborah D Stocken, Kathryn Carruthers, Harry Hemingway, Andrew T Yan, Shaun G Goodman, David Brieger, Derek P Chew, Chris P Gale

**Affiliations:** 1 Clinical Trials Research Unit, Leeds Institute for Clinical Trials Research, University of Leeds, Leeds, UK; 2 Royal Infirmary of Edinburgh, Edinburgh, UK; 3 Medical Statistics, London School of Hygiene and Tropical Medicine, London, UK; 4 Centre for Cardiovascular Science, University of Edinburgh, Edinburgh, UK; 5 Health Data Research UK London, UCL, London, UK; 6 Institute of Health Informatics, UCL, London, UK; 7 The National Institute for Health Research UCL Hospitals Biomedical Research Centre, UCL, London, UK; 8 St. Michael’s Hospital, Toronto, Canada; 9 The University of Sydney, Sydney, Australia; 10 Department of Cardiovascular Medicine, Flinders Medical Centre, Bedford Park, South Australia, Australia; 11 Leeds Institute of Cardiovascular and Metabolic Medicine, University of Leeds, Leeds, UK

**Keywords:** acute coronary syndrome, nsteacs, cluster randomised trial, guideline-indicated treatment, risk stratification, grace

## Abstract

**Introduction:**

For non-ST-segment elevation acute coronary syndrome (NSTEACS) there is a gap between the use of class I guideline recommended therapies and clinical practice. The Global Registry of Acute Coronary Events (GRACE) risk score is recommended in international guidelines for the risk stratification of NSTEACS, but its impact on adherence to guideline-indicated treatments and reducing adverse clinical outcomes is unknown. The objective of the UK GRACE Risk Score Intervention Study (UKGRIS) trial is to assess the effectiveness of the GRACE risk score tool and associated treatment recommendations on the use of guideline-indicated care and clinical outcomes.

**Methods and analysis:**

The UKGRIS, a parallel-group cluster randomised registry-based controlled trial, will allocate hospitals in a 1:1 ratio to manage NSTEACS by standard care or according to the GRACE risk score and associated international guidelines. UKGRIS will recruit a minimum of 3000 patients from at least 30 English National Health Service hospitals and collect healthcare data from national electronic health records. The co-primary endpoints are the use of guideline-indicated therapies, and the composite of cardiovascular death, non-fatal myocardial infarction, new onset heart failure hospitalisation or cardiovascular readmission at 12 months. Secondary endpoints include duration of inpatient hospital stay over 12 months, EQ-5D-5L responses and utilities, unscheduled revascularisation and the components of the composite endpoint over 12 months follow-up.

**Ethics and dissemination:**

The study has ethical approval (North East - Tyne & Wear South Research Ethics Committee reference: 14/NE/1180). Findings will be announced at relevant conferences and published in peer-reviewed journals in line with the funder’s open access policy.

**Trial registration number:**

ISRCTN29731761; Pre-results.

**Strengths and limitations of this study**

- UK GRACE Risk Score Intervention Study is a trial of patient management according to a risk model to reduce adverse patient clinical outcomes. This assessment of the effectiveness of risk stratification uses level one scientific methods.
- The design, intervention, patient population and outcomes are sufficiently similar to existing and planned international trials to facilitate individual participant data meta-analysis.
- The trial will use nationwide patient–level health records to enhance baseline and outcomes information derived from clinical records and patient questionnaires.
- Compliance with the assigned intervention at the cluster level requires monitoring, and clinical events cannot be centrally adjudicated, as they arise from electronic health records. As such, this is a pragmatic trial with results generalisable to the ‘real world’ clinical environment.

## Background

Non-ST-segment elevation acute coronary syndrome (NSTEACS) is a leading cause of disability, hospitalisation and death, and has major impacts on health economies.^1^ Since NSTEACS prognosis is determined by both baseline clinical risk and the use of evidence-based therapies, appropriately- stratified and effectively-delivered NSTEACS care has the potential to achieve cost-effective patient-centred treatment and improve clinical outcomes.^2–5^ For NSTEACS, however, there is a risk-treatment paradox whereby lower rather than higher risk patients are more likely to receive an invasive coronary strategy and more aggressive pharmacotherapies.^6–8^ The paradox is due to a number of factors including inaccurate and subjective risk assessment, under-recognition of the potential treatment benefits that higher risk cohorts may have, and cumulative missed care opportunities.^9–12^

Despite recommendation by national agencies such as the National Institute for Health and Care Excellence, the European Society of Cardiology and the American College of Cardiology (ACC)/American Heart Association (AHA),^2–5^ scoring systems which objectively characterise cardiovascular risk are not systematically applied to the management of NSTEACS.^12^ The Global Registry of Acute Coronary Events (GRACE) risk score tool is specifically designed for the risk stratification of patients with acute coronary syndrome (ACS), is aligned to treatment recommendations and, compared with other ACS risk scores, has superior discriminative performance.^13 14^ It was developed in an international registry programme to predict in-hospital and 6 month death or non-fatal myocardial infarction (MI) in a broad spectrum of ACS patients, and has since been derived and validated for these outcomes in over 42 000 patients with external validation in other cohorts.^15 16^ For clinical use, a simplified model was derived that predicts the risk of death alone, and the composite outcome of death or non-fatal MI, based on eight variables (age, heart rate, systolic blood pressure, Killip class, creatinine concentration, elevated biomarkers of myocardial injury, cardiac arrest on admission and ST-segment deviation). These factors conveyed more than 90% of the total risk and had a c-statistic of 0.81 for predicting death and 0.73 for predicting death or non-fatal MI from admission to 6 months after discharge.^17^ The GRACE risk score also shows good discriminatory performance for mortality at up to 2 years following discharge from hospital with ACS.^18^ The GRACE risk score has been updated (http://www.outcomes-umassmed.org/grace/) and shows good predictive accuracy of 1 year and 3 year mortality across a spectrum of ACS types.^19 20^

While evidence from registry data suggests that more comprehensive NSTEACS treatment is associated with improved outcome, there are no studies that have tested whether the prospective use of the GRACE risk score tool improves adherence to guideline recommendations for the management of NSTEACS and reduces adverse clinical outcomes.^8 21^ Complex behavioural interventions, such as the use of a risk score tool, have several interacting components. Typically, they present difficulties in (1) the standardisation of the design and delivery of the intervention, (2) their sensitivity to local context, (3) the organisational difficulty of applying experimental methods to service change and (4) the length and complexity of the causal chains linking the intervention with outcomes.^22^ It is not surprising, therefore, that some randomised controlled trials (RCTs) of complex interventions have failed to show improvements in outcomes, while others have succeeded.^23 24^

The UK is unique in that (1) national data are systematically and routinely collected for the populace regarding hospital healthcare utilisation and mortality, and for those admitted to hospitals with ACS information about cardiac investigations, pharmacotherapies and invasive coronary procedures^25^ and (2) the National Institute for Health Research through its Clinical Research Network supports National Health Service (NHS) hospitals to undertake research at scale. Thus, we aim to harness this national platform to undertake an efficient, pragmatic RCT to test the hypothesis that the use of the GRACE risk score tool will increase guideline-recommended treatment and decrease clinical events in NSTEACS patients.

## Methods and design

### Objectives

The UK GRACE Risk Score Intervention Study (UKGRIS; ISRCTN29731761, online supplementary file 1) will test the effectiveness of the use of the GRACE risk score tool by healthcare professionals on the delivery of guideline recommended care and clinical outcomes among patients hospitalised with NSTEACS. Co-primary objectives of the trial are:

1. To determine whether implementing the GRACE risk score increases the use of class I guideline-indicated therapies for the management of NSTEACS within the guideline recommended time, compared with current standard care;
2. To determine whether implementing the GRACE risk score reduces the composite endpoints of cardiovascular death, non-fatal MI, new onset heart failure and cardiovascular readmission at 12 months, compared with current standard care.

10.1136/bmjopen-2019-032165.supp1 Supplementary data

Secondary objectives are to determine whether implementing the GRACE risk score has an impact on:

1. Health-related quality of life at 12 months post-registration (measured by the EQ-5D-5L questionnaire);
2. Occurrence of unscheduled coronary revascularisation within the 12 months of initial presentation;
3. Duration of hospital stay within 12 months of initial presentation;
4. The individual components of the co-primary composite endpoint within 12 months.

### Trial design

This publication describes V.3.0 of the UKGRIS protocol, dated 12^th^ November 2018.

UKGRIS is a two-arm 1:1 parallel-group, open-label, multicentre, cluster-randomised, controlled registry-based superiority trial. UK hospitals are eligible to participate in UKGRIS if they are acute NHS hospitals participating in the Myocardial Ischaemia National Audit Project and willing to implement the GRACE risk score tool if randomised to that arm. Hospitals are ineligible if they are already routinely using the GRACE risk score tool prior to randomisation.

#### Randomisation and blinding

**Figure 1:**
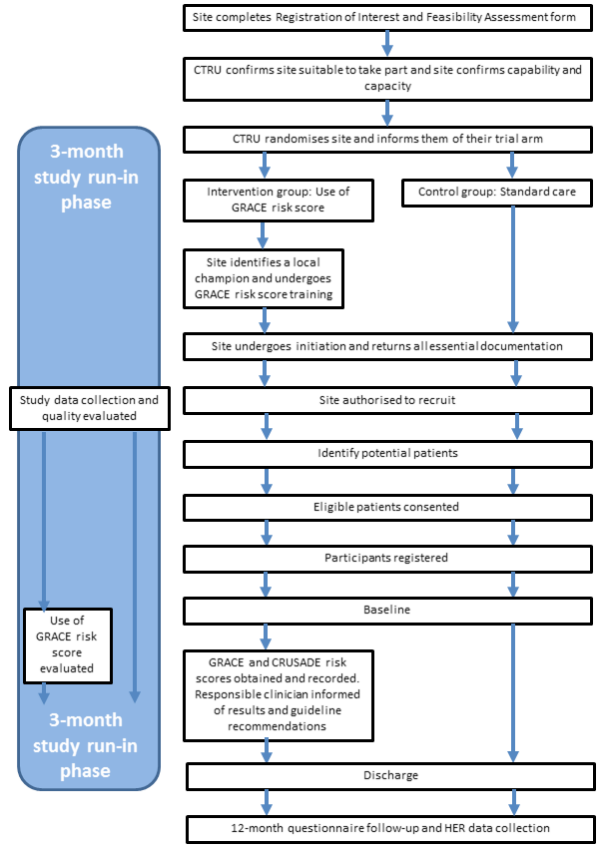
GRACE site recruitment and study run-in. CTRU, Leeds Clinical Trials Research Unit; GRACE, Global Registry of Acute Coronary Events.

Eligible hospitals (clusters) will be centrally randomised by the Leeds Clinical Trials Research Unit (CTRU) 1:1 to either GRACE risk score guided management or standard care using minimisation with a random element, aiming to ensure the cluster composition of each study arm is similar with respect to cluster-specific volume of patients hospitalised with ACS and primary percutaneous coronary intervention (PCI) capability. Site staff will not be blind to their assigned intervention, nor will recruited participants be blind to the intervention assigned to that site. Once a hospital has been randomised to the use of the GRACE risk score tool or to standard care, informed of their allocation and completed site initiation as required for their arm, a run-in phase will take place. During this time (up to 3 months for each site), the data collection and quality will be evaluated and the frequency of GRACE application in the intervention arm sites will be measured, as patients are recruited. (figure 1).

### Eligibility criteria

Participants will be recruited from the randomised hospital to which they present. Eligibility criteria are deliberately broad and pragmatic: participants must be aged 18 years and above, hospitalised with NSTEACS (either non-ST-elevation myocardial infarction (NSTEMI) or unstable angina) and provide written informed consent (box 1). They must not present with ST-elevation myocardial infarction, or with NSTEACS precipitated by a clear non-cardiovascular cause (for example, motor vehicle accident, trauma, severe gastrointestinal bleeding, perioperative/periprocedural myocardial infarction) or be previously enrolled in the trial. 

Box 1 **Full eligibility criteria for participants**

**Participant inclusion criteria**

1. Aged≥18 years

2. Symptoms consistent with acute cardiac ischaemia for >10 mins within 24 hours of presentation to hospital plus either or both of:

- one of (a,b,c);
- at least two of the High Risk Features (d)

(a) ECG changes:

- Transient ST-segment elevation of 0.5 mm in two or more contiguous leads;
- ST-segment depression of 0.5 mm in two or more contiguous leads;
- New T wave inversion of 1 mm in two or more contiguous leads;
- New Q waves (1/3 height of R wave or >0.04 s);
- New R wave>S wave in lead V1; or,
- New left bundle branch block

(b) Elevated cardiac biomarkers:

- Troponin T or I above the upper reference limit (URL);
- **creatine kinase**-muscle/brain (CK-MB) 2x URL; or,
- If there is no CK-MB available, then total CK greater than the local URL

(c) Documented coronary artery disease:

- History of myocardial infarction (MI) or angina;
- Congestive cardiac failure due to ischaemia;
- Resuscitated sudden cardiac death;
- Prior or new positive stress test with or without imaging;
- Prior or new, cardiac catheterisation, percutaneous coronary artery intervention or coronary artery bypass graft surgery documenting coronary artery disease

(d) **At least** two of the following High Risk features:

- Haemodynamic compromise (systolic blood pressure <90 mmHg and heart rate >100 bpm)
- Left ventricular systolic dysfunction (left ventricular ejection fraction <0.40);
- Presence of known diabetes mellitus
- Documentation of chronic kidney disease (estimated glomerular filtration rate <60mls/min/m^2^)

3. Willing and able to provide written informed consent

**Participant exclusion criteria**

1. Patients presenting at hospital due to an acute ST-segment elevation myocardial infarction
2. Patients presenting at hospital with an acute coronary syndrome accompanied with, or precipitate by significant comorbidity for example, motor vehicle accident, trauma, severe gastrointestinal bleeding
3. Perioperative or periprocedural MI
4. Patients already recruited into the study
5. Patients not living in the UK

The site Principal Investigator or a delegated member of the clinical research team will undertake the informed consent process. The participant will be approached within 12 hours of hospital admission (ideally in the emergency department or acute admission ward, whichever is closer to the time of hospitalisation), allowed time to consider the information and ask questions before deciding whether to participate. An optional oral consent pathway is available where participants will be provided with a shortened document describing the UKGRIS trial and after discussing the trial with the research nurse, can verbally consent to commence the care assigned to the admitting hospital. In hospitals allocated to GRACE, risk scores will be calculated and treatment recommendations made. Thereafter, the patient will be approached for full written informed consent. Participants who give oral consent at this point will be registered and complete the self-reported questionnaire and frailty score. Participants who give oral consent and refuse written informed consent will be excluded, and completed case record forms (CRFs) destroyed.

The informed consent process will request consent for data from all registeredpatients to be included in planned collaborative international analysis to investigate the GRACE risk score internationally, based on a planned individual patient data (IPD) meta-analysis of similar trials in Australia and Canada.^26^ The right of the participant to refuse participation without prejudice to future care will be respected, as will be the right to withdraw consent to further participation.

Efforts to ensure adequate and broad recruitment of all eligible patients in both arms through site research staff include: designation of a site ‘champion’, responsible for ensuring all staff are aware of the study and regular contacts to identify barriers to recruitment and/or trial compliance, trial posters, a twitter account (@UKGRIS_Trial).

### Trial status

The first site opened to recruitment on 13^th^ February, 2017, and the first patient was recruited on 13^th^ March, 2017. At the time of manuscript submission, 39 hospitals had been randomised to a study arm (of which 30 have registered at least one patient), and 2515 patients had consented to take part. Recruitment is expected to end on 31^st^ December, 2019, with end of follow-up ending on 31^st^ December, 2020, and completion of analysis by 30^th^ June, 2021.

### Governance

The trial is funded by the British Heart Foundation (grant reference CS/16/2/32145) and the sponsor is the University of Leeds (Leeds, UK). Neither the sponsor nor the funders are involved with study design, data collection, management, analysis or interpretation, nor will they have any influence on decisions relating to publication. The funders may review draft publications, but all final decisions will rest with the authors. The sponsor reserves the right to conduct periodic source data verification to monitor the integrity of the trial.

### Interventions

#### GRACE risk score tool

**Figure 2:**
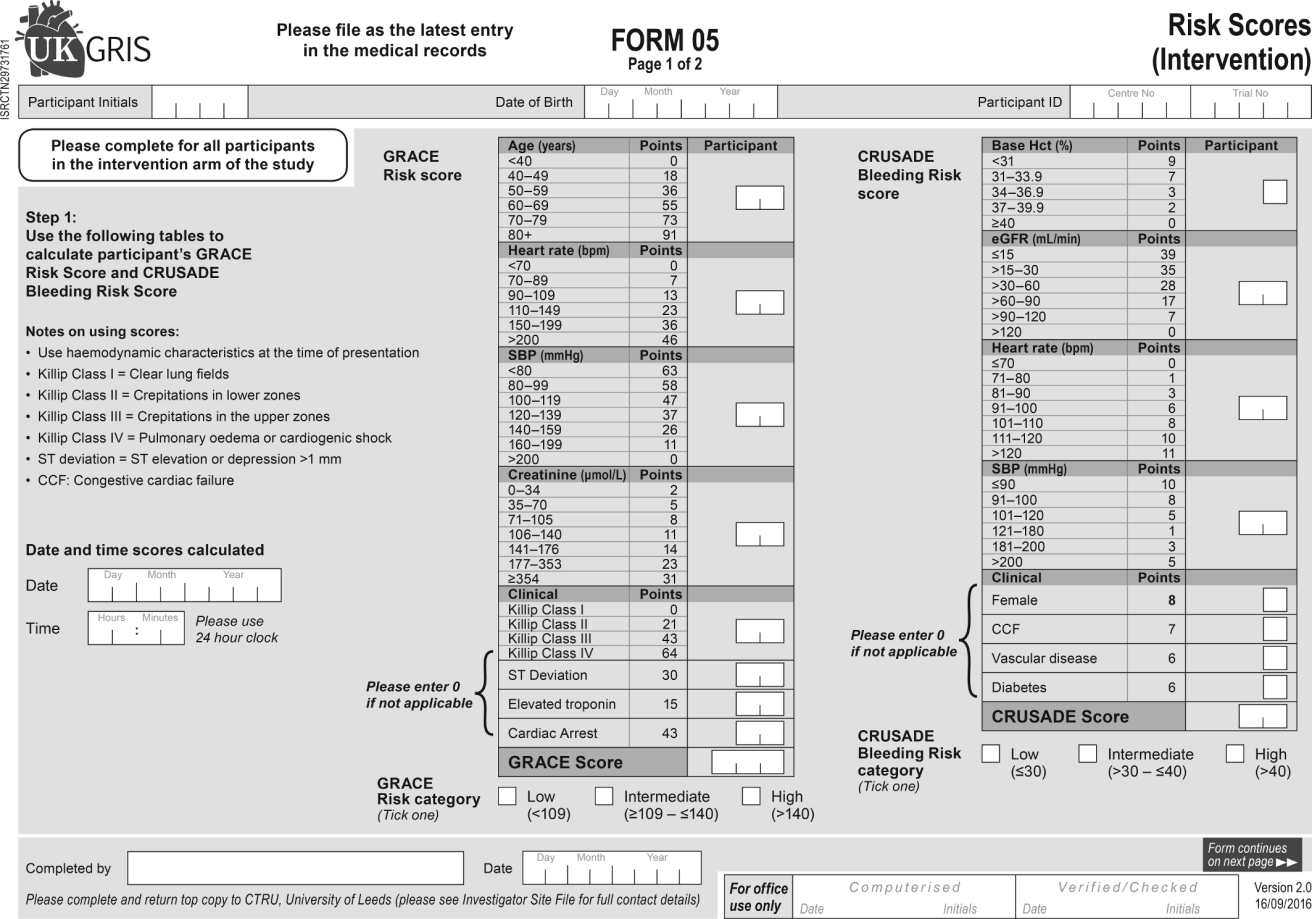
GRACE and CRUSADE risk scoring paper chart (completed by intervention sites only, and not distributed to control arm sites). CRUSADE, Can Rapid risk stratification of Unstable angina patients Suppress ADverse outcomes with Early implementation of the ACC/AHA Guidelines; CTRU, Leeds Clinical Trials Research Unit; eGFR, estimated glomerular filtration rate; GRACE, Global Registry of Acute Coronary Events; Hct, haematocrit; SBP, systolic blood pressure; UKGRIS, UK GRACE Risk Score Intervention Study.

**Figure 3:**
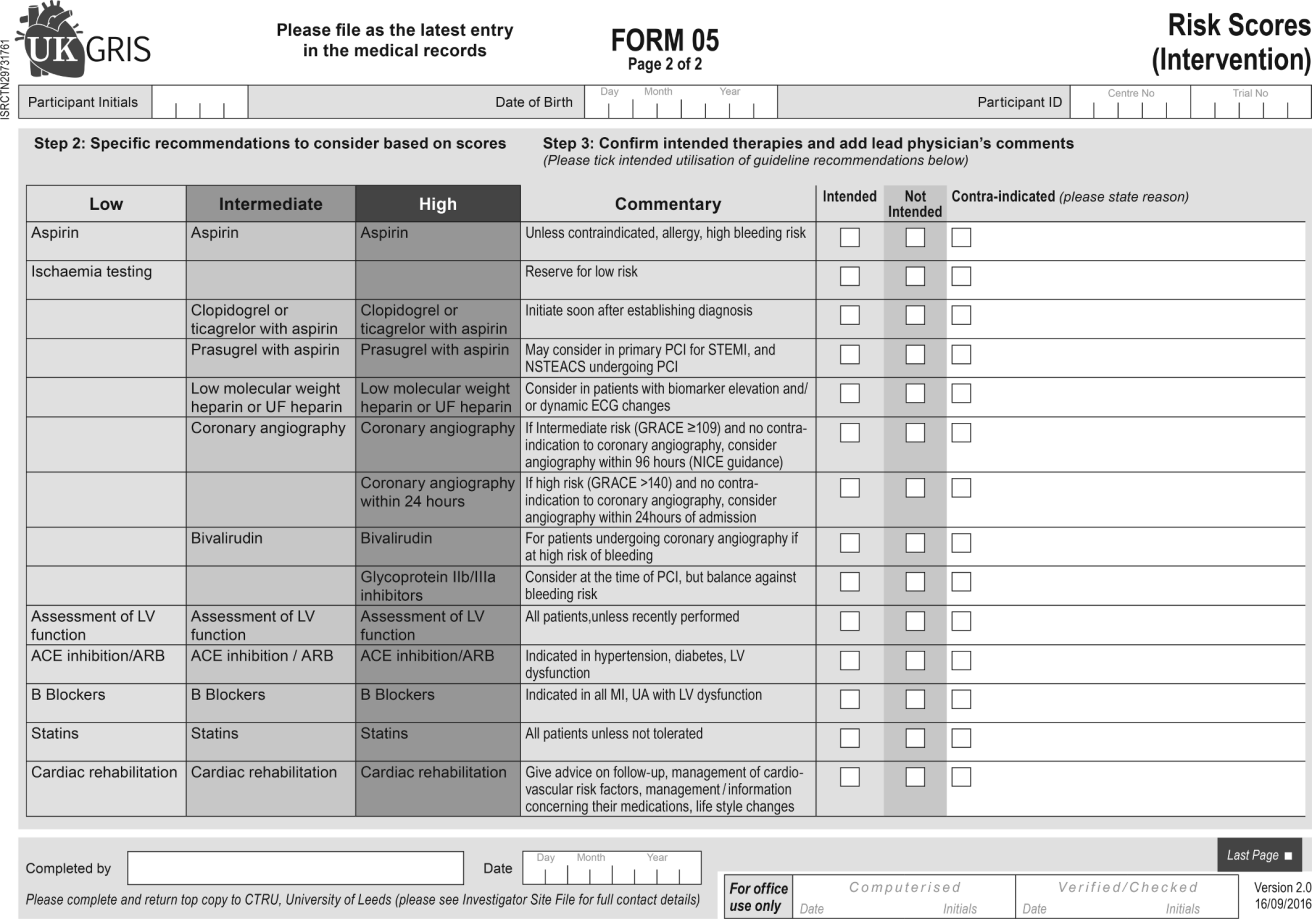
Associated guideline recommended therapies for GRACE risk score categories (completed by intervention sites only, not distributed to control arm sites). ACE, angiotensin-converting enzyme; ARB, angiotensin receptor blocker; CTRU, Leeds Clinical Trials Research Unit; GRACE, Global Registry of Acute Coronary Events; LV, left ventricular; MI, myocardial infarction; NICE, National Institute for Health and Care Excellence; NSTEACS, non-ST-segment elevation acute coronary syndrome; PCI, percutaneous coronary intervention; STEMI, ST-elevation myocardial infarction; UA, urinalysis; UF, unfractionated; UKGRIS, UK GRACE Risk Score Intervention Study.

Consenting participants recruited from hospitals in the intervention arm will receive care determined by staff trained to use the GRACE risk score tool and provided with a paper scoring table. Each consenting participant will have their risk score and corresponding 6 month mortality risk estimated by the appointed healthcare professional within 12 hours of hospitalisation. GRACE risk scores are categorised as ‘low’ (0 to 108), ‘intermediate’ (109 to 140) or ‘high’ (≥141), and the risk score nomogram (figure 2) will list recommended therapies (including pharmacological, invasive, non-invasive and behavioural interventions and tests) for patients based on their risk category (figure 3). At the same time as the GRACE risk score is calculated, sites will complete the CRUSADE bleeding risk score (Can Rapid risk stratification of Unstable angina patients Suppress ADverse outcomes with Early implementation of the ACC/AHA Guidelines).^27^ Both GRACE and CRUSADE will be completed using pen and paper scoring tables provided by the CTRU, online scoring applications and websites will not be used. Figure 2 illustrates the GRACE risk score nomogram as used in UKGRIS, while figure 3 illustrates the resulting recommendations arising from the scores obtained. Sites are invited to justify their decisions in declining to follow a recommendation on the grounds of contraindication. As part of the site initiation process, intervention sites will be informed as to how to complete the GRACE risk score paper nomogram, and follow its resulting recommendations. Sites will be monitored by telephone interviews with the ‘site champion’ throughout recruitment to ensure that GRACE and CRUSADE scoring is still routinely used for managing patients with NSTEACS. If GRACE sites stop routinely using the GRACE and CRUSADE risk scores, they will be asked to resume their use, and to cease recruitment to the UKGRIS trial if they fail to do so.

#### Standard care

Hospitals randomised to standard care will treat patients according to local practice (without using the GRACE risk score tool and associated clinical prompts) (figure 1). Sites will be monitored by telephone interviews with the ‘site champion’ throughout recruitment to ensure that processes for managing patients with NSTEACS are unchanged and that the management according to the GRACE risk score has not been systematically implemented due to a change in hospital policy. Standard care-assigned sites should not be routinely using the GRACE risk score nor the CRUSADE bleeding risk score to manage NSTEACS patients while taking part in the trial. Standard care sites implementing either scoring system during the study (as identified during regular site monitoring updates) will be asked to cease use and will stop recruitment to the UKGRIS trial if routine use continues.

### Outcome measures

There are two co-primary endpoints: (i) the uptake of class I guideline-indicated therapies (specifically, the 11 listed in online supplementary file 2) and (ii) the composite of cardiovascular death, non-fatal myocardial infarction, hospitalisation for new onset heart failure or cardiovascular readmission, at 12 months (table 1, with subsequently derived outcome measures in table 2). Secondary endpoints are total duration of inpatient stay, quality of life based on EQ-5D-5L^28^ responses and utilities, unscheduled coronary revascularisation and the components of the composite endpoint over 12 months follow-up.

10.1136/bmjopen-2019-032165.supp2 Supplementary data

**Table 1:** Schematic of assessments

TIME POINT*	Cluster identification	Cluster allocation	Patient identification	Patient registration	Post-allocation		
	*-t_3_*	*-t_2_*	-t_1_	*0*	*t_A_*	*t_D_*	***12*** m
CLUSTER ENROLMENT:							
Eligibility screen	X						
Essential documents	X						
Randomisation		X					
Site initiation		X					
PATIENT ENROLMENT:							
Eligibility			X				
Informed consent			X				
Registration				X			
INTERVENTIONS:							
GRACE/CRUSADE risk scoring (X) and subsequent care					X		
Standard care					X		
ASSESSMENTS:							
Baseline assessments*				X			
EQ-5D-5L				X			X
Edmonton frailty score				X			
Numbers of class I guideline indicated therapies					X		
Numbers of therapies received						X	X
Date of discharge						X	
Final diagnosis						X	
Medications prescribed acutely during hospital stay or on discharge						X	
In-hospital cardiovascular procedures						X	
In-hospital outcomes						X	
Other cardiac investigations and treatments						X	
Health advice given						X	
Hospitalisations							X
Deaths							X
Unscheduled revascularisations							X

*Time points: -t_3_, -t_2_, -t_1_ are three arbitrary time points that occur at any time (but in the given order) prior to individual patient enrolment at time 0. Post registration, t_A_ and t_D_ are the time points at which a participant is assessed according to the cluster-randomised strategy and discharged from hospital. Subsequent follow-up is at 12 months.

†Baseline assessments are: age, sex, ethnicity, height, weight, date and time of symptoms onset and admission to hospital, ECG ST-segment deviation, heart rate on admission, systolic blood pressure on admission, Killip class, diuretic usage, creatinine, troponin (date, time and elevated), cardiac arrest occurrence, haematocrit, peripheral vascular disease, diabetes mellitus, congestive cardiac failure.

CRUSADE, Can Rapid risk stratification of Unstable angina patients Suppress ADverse outcomes with Early implementation of the ACC/AHA Guidelines; GRACE, Global Registry of Acute Coronary Events.

**Table 2:** Definitions of UKGRIS outcome measures and primary analyses

Outcome measure	Assessed at	Analysis method
**Co-primary**		
(1) Number of class I guideline indicated processes received per patient (see online supplementary file 2 for full description)	Discharge	Logistic regression, modelling agreement between eligibility and receipt of process as function of treatment arm, including fixed effects for minimisation factors, random hospital effect and random patient effect.
(2) Time from registration until first occurrence of one of the following * cardiac death, * new onset heart failure, * non-fatal MI, or * cardiovascular hospitalisation	12 months post registration	Kaplan-Meier survival estimates; appropriate time-to-event regression (eg, proportional hazards, flexible parametric model^47^) including fixed effects for minimisation factors and random hospital effect, (sensitivity analysis: also accounts for competing risks of non-cardiac death and withdrawal from follow-up.)
**Secondary**		
(1) Health-related quality of life (EQ-5D-5L utility)	12 months post registration	Linear regression, (with data transformed, if necessary) including fixed effects for minimisation factors and baseline value with random hospital effect.
(2) Number of days in hospital within 12 months	12 months post registration	Linear regression (with data transformed, if necessary) including fixed effects for minimisation factors and random hospital effect.
(3) Time until first occurrence of each component of the composite co-primary endpoint (co-primary (2)). Performed separately for each of the four components.	12 months post registration	Kaplan-Meier survival estimates; time-to-event regression including fixed effects for minimisation factors and random hospital effect, accounting for (sensitivity analysis: competing risks of non-cardiac death and withdrawal from follow-up.)
(4) Unscheduled revascularisations. Any admission (1=Yes, 0=No) for an unplanned PCI or CABG within 12 months of registration.	12 months post registration	Logistic regression, including fixed effects for minimisation factors with random hospital effect.

CABG, coronary artery bypass graft; MI, myocardial infarction; PCI, percutaneous coronary intervention; UKGRIS, UK GRACE Risk Score Intervention Study.

### Data collection

Potential participants who do not take part (either ineligible or non-consenting) will be recorded on non-registration logs recording age, sex, ethnicity and reason for patient non-registration. These will be collected by local investigators and returned to the CTRU. Baseline variables for registered participants will be collected on paper CRFs by site staff, who will keep copies and return originals to the CTRU (except for consent forms which are kept at site and copies sent to the CTRU). GRACE and CRUSADE risk scores will be estimated for each participant at each participating hospital site randomised to use the GRACE risk score tool and recorded on CRFs. Since the paper nomogram approach is also being used in similar international trials, scoring via mobile phone apps or web-based calculators will not be permitted. Details of medications and tests prescribed and received during hospitalisation will be recorded. At baseline, the participants’ Edmonton frailty score^29^ will be assessed and all participants will complete the EQ-5D-5L questionnaire. At 12 months, surviving participants will be asked to complete and return a further EQ-5D-5L by post.

Follow-up data for hospital healthcare utilisation, and dates and causes of death will be collected using multi-source national electronic health records (EHRs). This will include data capture from the National Institute for Cardiovascular Outcomes Research^25^ suite of nationwide cardiovascular registries,^30 31^ Hospital Episode Statistics,^32^ Office for National Statistics and the National Audit of Cardiac Rehabilitation database.^33^ To obtain records, the CTRU will provide the patient identifiers to the responsible organisations (with patient consent obtained for this data transfer) who will link the identifiers to their records according to their own deterministic algorithms and return to the CTRU the relevant records via secure file transfer or data interrogation system. The occurrence of clinical events will not be centrally adjudicated, since the EHR do not include access to the detailed hospital notes. We will investigate the effect of discrepancies between the EHR and the data reported on the CRFs, on the trial results in a sensitivity analysis.

Data received via CRFs will be entered onto a dedicated CTRU database (MACRO, InferMed) which will include validations to identify missing or discrepant data. The trial statistician will download accumulated data to identify discrepant data items for investigation. Data received via EHR will be automatically uploaded to the MACRO database via a data import system.

### Statistical methods

#### Sample size

A minimum of 30 clusters, each recruiting 100 participants (ie, 3000 in total) will give 80% power to detect clinically relevant differences in the co-primary endpoints with two-sided 5% significance tests (see sections 7.2.1 and 7.2.3 of^34^). For the proportion of class I guideline-recommended therapies implemented, this assumes 95%^35^ of recommended therapies implemented in the control arm and will detect an absolute increase of 3% to 98% in the GRACE arm, with an assumed coefficient of variation (CV) in cluster outcomes of 0.02. For time to first cardiac outcome, a fixed recruitment of 15 clusters of 100 patients per arm gives 80% power to detect a difference in 12 month rates of 13% and 10.4% between standard care and GRACE arms, assuming a mean follow-up of 27 months and CV of cluster event rates of 0.05. Both endpoints require 14 clusters of 100 patients in each arm to have 80% power, though no adjustment for multiple testing was made. Recruiting 15 clusters per arm allows for attrition of 7% to 10%. For more details, see online supplementary file 3. Additional sites have been and will be opened to ensure that target recruitment is achieved, and to safeguard study power should these assumptions not be met.

10.1136/bmjopen-2019-032165.supp3 Supplementary data

#### Analysis methods

A full statistical analysis plan will be finalised prior to any comparative analyses, following the guidelines of Gamble *et al*.^36^ The statistical analysis plan will describe the derivation of outcomes, define analysis populations and detail the analyses of each outcome measure. In brief, analyses will take the form of generalised linear models that include fixed effects for the minimisation/stratification factors and random effects for the recruiting hospitals (the random effects distribution with the best statistical fit will be used for analysis) to reflect the cluster-randomised nature of the trial (table 2). The derivation of the primary outcome measure is defined in online supplementary file 2. Additional exploratory subgroup analyses are planned for the primary endpoint: analysing guideline uptake according to intervention type (invasive, non-invasive, behavioural and pharmacotherapies); subgroup effects will investigate interaction between treatment arm and age, gender, diabetes, heart failure, initial hospital type, frailty, comorbidity and estimated bleeding risks, among others. Analyses will use the full analysis set comprising all registered participants analysed according to their randomised hospital as intention to treat (patients will only be excluded if informed consent was not given, or if participants request that their data not be used in any analysis). Further supporting randomisation-respecting efficacy analyses may be considered, in particular, a mediation analysis^37^ to estimate the net direct and net indirect effects of a non-NSTEACS final diagnosis on receiving class I guideline therapies. The statistical analysis plan will include an agreed strategy for imputing missing data items (using multiple imputation, where appropriate^38 39^), including handling incomplete EHR on cardiovascular registries and other data sources to determine whether absent records indicate no events.

### Monitoring

Oversight of the trial will be the responsibility of the independent Trial Steering Committee (TSC), comprising two independent cardiologists, an independent statistician and one independent patient representative. An independent Data Monitoring and Ethics Committee (DMEC) will meet to review accumulating safety and compliance data from the UKGRIS trial. The members will comprise an independent statistician and three independent cardiologists. There are no planned interim analyses of efficacy for the UKGRIS trial. The members of the Trial Management Group, the DMEC and the TSC are listed in online supplementary file 4.

10.1136/bmjopen-2019-032165.supp4 Supplementary data

Given that the intervention carries minimal risk to participants, serious and non-serious unrelated or expected adverse events will not be reported. Instead, participant primary and secondary outcomes at the time of discharge from hospital (or death if it occurs during hospitalisation) until 12 months will be reviewed by the DMEC at a site level using CRFs and EHRs. All related and unexpected serious adverse events occurring from the date of consent up to 3 months following admission will be sent to the CTRU within 24 hours of the clinical research staff becoming aware of the event. Changes to the protocol will be provided to the Research Ethics Committee for review. On receipt of favourable opinion, new versions of the protocol will be communicated to sites for implementation. Sites randomised to routinely employ the GRACE risk score tool will be able to note where particular guideline-recommended therapies are deemed not appropriate. All sites will be able to report that guideline-recommended invasive strategies are not appropriate.

### Ethics and dissemination

A favourable opinion was provided by the North East - Tyne & Wear South Research Ethics Committee on 31^st^ October, 2016, (reference 14/NE/1180). Trial results will be disseminated at relevant conferences and published in peer-reviewed journals. Authorship will be decided according to ICMJE guidelines as to qualifying contributions, and the primary results manuscript jointly drafted by the Chief Investigator and the trial methodologists before circulating to remaining co-authors.

### Patient and public involvement

During the initial design stage, the applicants consulted the patient and public involvement group associated with the co-ordinating centre to inform the study design, particularly with regard to the participant burden, so as to reduce loss to follow-up. The Trial Steering Committee includes a patient representative. We have also consulted patient representatives as to the design of covering letters used in our 12 month mailed questionnaire follow-up.

## Discussion

International guidelines recommend the use of the GRACE risk score for stratification of patients hospitalised with NSTEACS such that they may receive evidence-based care according to their estimated risk of future ischaemic events.^2 3 5^ While observational studies have validated the GRACE risk score across diverse populations and for a range of clinical outcomes,^40^ and shown that a failure to follow NSTEMI guideline recommendations leads to excess mortality,^41^ there are no randomised data investigating whether the routine use of the GRACE risk score tool improves NSTEACS care and reduces subsequent cardiovascular events.

The UKGRIS trial will determine whether the systematic use of the GRACE risk score increases the use of class I guideline-indicated therapies for the management of NSTEACS and reduces a composite cardiovascular endpoint at 12 months compared with current standard care. UKGRIS will encompass multisource national EHRs for data capture of the primary endpoints. Moreover, UKGRIS will form part of an international collaborative consortium and run in parallel with trials with harmonised protocols in Australia^26^ and Canada. While the UKGRIS study will focus on impacts on clinical- and cost-effectiveness for a contemporary UK population, a harmonised protocol across the three international trials will facilitate IPD meta-analysis to improve precision of the estimated reduction in mortality and other cardiovascular events.

Optimising risk assessment of hospitalised NSTEACS may help reduce inequalities in the provision of care. Serving as a healthcare prompt at point of entry into the health service system, the GRACE risk score and associated international guideline recommendations for the management of NSTEACS may reduce the risk–treatment paradox. Recent observational data suggest that the decline in mortality over time among NSTEMI patients was significantly associated with the use of an invasive coronary strategy and the wider application to higher risk cases,^42^ and the impact of guideline-indicated treatments for NSTEMI persists for many years. Extending this initiative to undertreated higher risk NSTEACS may further improve clinical outcomes. Moreover, when compared with objective risk assessment, physician-estimated risk offers less discriminatory performance and is associated with lower rates of PCI among higher risk patients.^14 43 44^

UKGRIS follows the Medical Research Council framework for the development and evaluation of complex behavioural interventions.^45^ A complex behavioural intervention aiming to change health behaviour contains several interacting components. For UKGRIS, the intervention is the use of a risk score tool, reference to an internationally recommended algorithm for the management of NSTEACS and informing healthcare professionals of the results of the risk calculation and associated guideline-indications for treatment. Moreover, the GRACE risk score comprises components such as age and blood pressure which are predictors of adverse outcomes; a clinician may, consciously or otherwise, base care management decisions on the presence of these factors, without resorting to a formalised risk scoring system. Studies of complex behavioural interventions may be criticised because they do not determine how and why interventions work.^46^ Thus, UKGRIS will collect information as to why guideline recommendations are not followed and data concerning frailty (a possible justification for non-receipt of evidence-based care).

UKGRIS will collect clinical data from a range of national EHRs. Limitations inherent in this approach include non-capture of all data relating to the disease (data missing by design), missing and corrupt data as recorded in routine national databases, time lag of data acquisition, non-consent for data capture, contamination and lack of fidelity. UKGRIS aims to be a streamlined and pragmatical registry-based trial, answering finite questions efficiently. As such, *a priori* endpoints were designed around the availability of data (for example, hospitalised rather than all new heart failure diagnoses), and key safety outcomes (mortality) will be requested from the UK Office for National Statistics. Missing and corrupt data are infrequent in national administrative database and for clinical registries we will augment data collection through the CRFs. We assume data anomalies will be equally balanced between intervention and control arms through randomisation. To reduce the potential for within-site contamination of the intervention, UKGRIS will randomise to the intervention at the hospital level, and treatment arm fidelity will be monitored and maintained by regular contact with each site. We should acknowledge that clinicians at all sites may improve their individual guideline implementation rates due to the Hawthorne effect of taking part in the study. Existing registry data for patients not taking part may indicate how GRACE trial performance differs from ‘real-world’ practice.

## Conclusions

Following hospital presentation with NSTEACS, subsequent CV morbidity and mortality rates are high and there is a gap between international recommendations for treatment and clinical practice. UKGRIS, a registry-based cluster-randomised controlled trial will determine whether an integrated behavioural intervention (the routine use of the GRACE risk score tool and associated management guidance) increases the use of class I guideline-indicated therapies for the management of NSTEACS and reduces a composite cardiovascular endpoint at 12 months, compared with current standard care. Though UKGRIS will, on its own, provide evidence of the value of GRACE risk implementation for NSTEACS cases in the UK, the opportunity for an international IPD meta-analysis means that the benefits of the UKGRIS study will reach much further.

## Supplementary Material

Reviewer comments

## References

[1] Roffi M, Patrono C, Collet J-P, et al 2015 ESC Guidelines for the management of acute coronary syndromes in patients presenting without persistent ST-segment elevation. Eur Heart J 2016;37:267–315. 10.1093/eurheartj/ehv320 26320110

[2] Jneid H, Anderson JL, Wright RS, et al 2012 ACCF/AHA Focused Update of the Guideline for the Management of Patients With Unstable Angina/Non–ST-Elevation Myocardial Infarction (Updating the 2007 Guideline and Replacing the 2011 Focused Update). A Report of the American College of Cardiology Foundation/American Heart Association Task Force on Practice Guidelines 2012;126:875–910. 10.1161/CIR.0b013e318256f1e0 22800849

[3] Steg PG, James SK, Atar D, et al ESC Guidelines for the management of acute myocardial infarction in patients presenting with ST-segment elevation. The Task Force on the management of ST-segment elevation acute myocardial infarction of the European Society of Cardiology 2012;33:2569–619. 10.1093/eurheartj/ehs215 22922416

[4] SIGN. Acute coronary syndromes: a national clinical guideline. (93). Edinburgh, UK: Scottish Intercollegiate Guidelines Network, 2007.

[5] NICE. Unstable angina and NSTEMI: the early management of unstable angina and non ST-segment-elevation myocardial infarction. (Clinical Guideline 94), 2010. 21977549

[6] Yan AT, Yan RT, Tan M, et al Management patterns in relation to risk stratification among patients with non-ST elevation acute coronary syndromes. Arch Intern Med 2007;167:1009–16. 10.1001/archinte.167.10.1009 17533203

[7] Simms AD, Batin PD, Weston CF, et al An evaluation of composite indicators of hospital acute myocardial infarction care: a study of 136,392 patients from the Myocardial Ischaemia National Audit Project. Int J Cardiol 2013;170:81–7. 10.1016/j.ijcard.2013.10.027 24182669

[8] Fox KA, Anderson FA, Dabbous OH, et al Intervention in acute coronary syndromes: do patients undergo intervention on the basis of their risk characteristics? The Global Registry of Acute Coronary Events (GRACE). Heart 2007;93:177–82. 10.1136/hrt.2005.084830 16757543 PMC1861403

[9] Peterson ED, Roe MT, Mulgund J, et al Association between hospital process performance and outcomes among patients with acute coronary syndromes. JAMA 2006;295:1912–20. 10.1001/jama.295.16.1912 16639050

[10] Alexander KP, Chen AY, Roe MT, et al Excess dosing of antiplatelet and antithrombin agents in the treatment of non-ST-segment elevation acute coronary syndromes. JAMA 2005;294:3108–16. 10.1001/jama.294.24.3108 16380591

[11] Califf RM, Peterson ED, Gibbons RJ, et al Integrating quality into the cycle of therapeutic development. J Am Coll Cardiol 2002;40:1895–901. 10.1016/S0735-1097(02)02537-8 12475447

[12] Bing R, Goodman SG, Yan AT, et al Use of clinical risk stratification in non-ST elevation acute coronary syndromes: an analysis from the CONCORDANCE registry. Eur Heart J Qual Care Clin Outcomes 2018;4:309–17. 10.1093/ehjqcco/qcy002 29438470

[13] Gale CP, Manda SO, Weston CF, et al Evaluation of risk scores for risk stratification of acute coronary syndromes in the Myocardial Infarction National Audit Project (MINAP) database. Heart 2009;95:221–7. 10.1136/hrt.2008.144022 18467355

[14] Yan AT, Yan RT, Tan M, et al Risk scores for risk stratification in acute coronary syndromes: useful but simpler is not necessarily better. Eur Heart J 2007;28:1072–8. 10.1093/eurheartj/ehm004 17437970

[15] Granger CB, Goldberg RJ, Dabbous O, et al Predictors of hospital mortality in the global registry of acute coronary events. Arch Intern Med 2003;163:2345–53. 10.1001/archinte.163.19.2345 14581255

[16] Eagle KA, Lim MJ, Dabbous OH, et al A validated prediction model for all forms of acute coronary syndrome: estimating the risk of 6-month postdischarge death in an international registry. JAMA 2004;291:2727–33. 10.1001/jama.291.22.2727 15187054

[17] Fox KA, Dabbous OH, Goldberg RJ, et al Prediction of risk of death and myocardial infarction in the six months after presentation with acute coronary syndrome: prospective multinational observational study (GRACE). BMJ 2006;333:1091 10.1136/bmj.38985.646481.55 17032691 PMC1661748

[18] Alnasser SM, Huang W, Gore JM, et al Late consequences of acute coronary syndromes: global registry of acute coronary events (GRACE) Follow-up. Am J Med 2015;128:766–75. 10.1016/j.amjmed.2014.12.007 25554379

[19] Huang W, FitzGerald G, Goldberg RJ, et al Performance of the GRACE Risk Score 2.0 Simplified Algorithm for Predicting 1-Year Death After Hospitalization for an Acute Coronary Syndrome in a Contemporary Multiracial Cohort. Am J Cardiol 2016;118:1105–10. 10.1016/j.amjcard.2016.07.029 27561191 PMC5050116

[20] Kaa F, FitzGerald G, Puymirat E, et al Should patients with acute coronary disease be stratified for management according to their risk? Derivation, external validation and outcomes using the updated GRACE risk score. BMJ Open 2014;4. 10.1136/bmjopen-2013-004425 PMC3931985 24561498

[21] Bawamia B, Mehran R, Qiu W, et al Risk scores in acute coronary syndrome and percutaneous coronary intervention: a review. Am Heart J 2013;165:441–50. 10.1016/j.ahj.2012.12.020 23537960

[22] Connors AF, Dawson NV, Desbiens NA, et al A controlled trial to improve care for seriously ill hospitalized patients. The study to understand prognoses and preferences for outcomes and risks of treatments (SUPPORT). The SUPPORT Principal Investigators. JAMA 1995;274:1591–8. 7474243

[23] Tiessen AH, Smit AJ, Broer J, et al Randomized controlled trial on cardiovascular risk management by practice nurses supported by self-monitoring in primary care. BMC Fam Pract 2012;13:90 10.1186/1471-2296-13-90 22947269 PMC3503756

[24] Ijzelenberg W, Hellemans IM, van Tulder MW, et al The effect of a comprehensive lifestyle intervention on cardiovascular risk factors in pharmacologically treated patients with stable cardiovascular disease compared to usual care: a randomised controlled trial. BMC Cardiovasc Disord 2012;12:71 10.1186/1471-2261-12-71 22962863 PMC3479017

[25] Gale CP, Weston C, Denaxas S, et al Engaging with the clinical data transparency initiative: a view from the National Institute for Cardiovascular Outcomes Research (NICOR). Heart 2012;98:1040–3. 10.1136/heartjnl-2012-302469 22739635

[26] Chew DP, Astley CM, Luker H, et al A cluster randomized trial of objective risk assessment versus standard care for acute coronary syndromes: Rationale and design of the Australian GRACE Risk score Intervention Study (AGRIS). Am Heart J 2015;170:995–1004. 10.1016/j.ahj.2015.07.032 26542510

[27] Subherwal S, Bach RG, Chen AY, et al Baseline Risk of Major Bleeding in Non–ST-Segment–Elevation Myocardial Infarction. The CRUSADE (Can Rapid risk stratification of Unstable angina patients Suppress ADverse outcomes with Early implementation of the ACC/AHA guidelines) Bleeding Score 2009;119:1873–82. 10.1161/CIRCULATIONAHA.108.828541 PMC3767035 19332461

[28] Janssen MF, Pickard AS, Golicki D, et al Measurement properties of the EQ-5D-5L compared to the EQ-5D-3L across eight patient groups: a multi-country study. Qual Life Res 2013;22:1717–27. 10.1007/s11136-012-0322-4 23184421 PMC3764313

[29] Rolfson DB, Majumdar SR, Tsuyuki RT, et al Validity and reliability of the Edmonton Frail Scale. Age Ageing 2006;35:526–9. 10.1093/ageing/afl041 16757522 PMC5955195

[30] Herrett E, Smeeth L, Walker L, et al The Myocardial Ischaemia National Audit Project (MINAP). Heart 2010;96:1264–7. 10.1136/hrt.2009.192328 20659944 PMC3505836

[31] Ludman PF British Cardiovascular Intervention Society. British Cardiovascular Intervention Society Registry for audit and quality assessment of percutaneous coronary interventions in the United Kingdom. Heart 2011;97:1293–7. 10.1136/heartjnl-2011-300299 21719554

[32] NHS Digital. Hospital Episode Statistics. http://content.digital.nhs.uk/hes (Accessed 5th May 2017).

[33] British Heart Foundation (BHF). National audit of cardiac rehabilitation. http://www.cardiacrehabilitation.org.uk/ (accessed 5th May 2017).

[34] Hayes RJ ML Cluster randomised trials. Boca Raton, FL: Chapman and Hall/CRC, 2009:105–28.

[35] Simms AD, Baxter PD, Cattle BA, et al An assessment of composite measures of hospital performance and associated mortality for patients with acute myocardial infarction. Analysis of individual hospital performance and outcome for the National Institute for Cardiovascular Outcomes Research (NICOR). Eur Heart J Acute Cardiovasc Care 2013;2:9–18. 10.1177/2048872612469132 24062929 PMC3760578

[36] Gamble C, Krishan A, Stocken D, et al Guidelines for the Content of Statistical Analysis Plans in Clinical Trials. JAMA 2017;318:2337–43. 10.1001/jama.2017.18556 29260229

[37] VanderWeele T Explanation in causal inference: methods for mediation and interaction: Oxford University Press, 2015.

[38] Cattle BA, Baxter PD, Greenwood DC, et al Multiple imputation for completion of a national clinical audit dataset. Stat Med 2011;30:2736–53. 10.1002/sim.4314 21786284

[39] White IR, Royston P, Wood AM Multiple imputation using chained equations: Issues and guidance for practice. Stat Med 2011;30:377–99. 10.1002/sim.4067 21225900

[40] Simms AD, Reynolds S, Pieper K, et al Evaluation of the NICE mini-GRACE risk scores for acute myocardial infarction using the Myocardial Ischaemia National Audit Project (MINAP) 2003-2009: National Institute for Cardiovascular Outcomes Research (NICOR). Heart 2013;99:35–40. 10.1136/heartjnl-2012-302632 23002253

[41] Dondo TB, Hall M, Timmis AD, et al Excess mortality and guideline-indicated care following non-ST-elevation myocardial infarction. Eur Heart J Acute Cardiovasc Care 2017;6:2048872616647705 10.1177/2048872616647705 27142174

[42] Hall M, Dondo TB, Yan AT, et al Association of clinical factors and therapeutic strategies with improvements in survival following non-st-elevation myocardial infarction, 2003-2013. JAMA 2016;316:1073–82. 10.1001/jama.2016.10766 27574717

[43] Chew DP, Junbo G, Parsonage W, et al Perceived risk of ischemic and bleeding events in acute coronary syndromes. Circ Cardiovasc Qual Outcomes 2013;6:299–308. 10.1161/CIRCOUTCOMES.111.000072 23652735

[44] Chew DP, Juergens C, French J, et al An examination of clinical intuition in risk assessment among acute coronary syndromes patients: observations from a prospective multi-center international observational registry. Int J Cardiol 2014;171:209–16. 10.1016/j.ijcard.2013.12.010 24380497

[45] Craig P, Dieppe P, Macintyre S, et al Developing and evaluating complex interventions: new guidance, 2006.

[46] Grant A, Treweek S, Dreischulte T, et al Process evaluations for cluster-randomised trials of complex interventions: a proposed framework for design and reporting. Trials 2013;14:15 10.1186/1745-6215-14-15 23311722 PMC3600672

[47] Royston P, Parmar MK Flexible parametric proportional-hazards and proportional-odds models for censored survival data, with application to prognostic modelling and estimation of treatment effects. Stat Med 2002;21:2175–97. 10.1002/sim.1203 12210632

