# Supplementary material for: Evaluation of the impact of the GRACE risk score on the management and outcome of patients hospitalised with non-ST elevation acute coronary syndrome in the UK: protocol of the UKGRIS cluster-randomised registry-based trial
Source: BMJ Open. 2019 Sep 5;9(9):e032165. doi: 10.1136/bmjopen-2019-032165 (PMC6731819; doi:10.1136/bmjopen-2019-032165)
Supplement: Supplementary data [file bmjopen-2019-032165supp001.pdf]

ONLINE SUPPLEMENTARY MATERIAL

Supplementary file 1 – WHO Trial Registration Dataset

Title

Evaluation of the impact of the GRACE risk score on the management and outcome of patients hospitalised with non-ST elevation acute coronary syndrome in the UK: protocol of the UKGRIS cluster-randomised registry-based trial.

Colin C. Everett, Keith A. A. Fox, Catherine Reynolds, Catherine Fernandez, Linda D. Sharples, Deborah D. Stocken, Kathryn Carruthers, Harry Hemingway, Andrew T. Yan, Shaun G. Goodman, David Brieger, Derek P. Chew, Chris P. Gale.

Supplementary File 1: WHO Trial Registration Dataset

Primary Registry and Trial Identifying Number	ISRCTN29731761
Date of Registration in Primary Registry	12th January 2017
Secondary Identifying Numbers	Research Ethics Committee ref: 4/NE/1180.<br>Funder's grant ref: CS/16/2/32145
Source(s) of Monetary or Material Support	British Heart Foundation, United Kingdom.
Primary Sponsor	University of Leeds, United Kingdom.
Secondary Sponsor(s)	None
Contact for Public Queries	Prof Chris P. Gale
Contact for Scientific Queries	Prof Chris P. Gale
Public Title	UK GRACE Risk Score Intervention Study (UKGRIS)
Scientific Title	UK GRACE Risk Score Intervention Study (UKGRIS)
Countries of Recruitment	United Kingdom only
Health Condition(s) or Problem(s) Studied	Non ST-segment-Elevation Acute Coronary Syndrome (NSTEACS)
Intervention(s)	Management by GRACE risk score or management by standard care (site practice)
Key Inclusion and Exclusion Criteria	See Table 1 of main publication.
Study Type	Parallel-group, open-label, cluster randomised controlled trial
Date of First Enrolment	9th March 2017
Target Sample Size	3000 patients from 30 clusters
Recruitment Status	Open to recruitment
Primary Outcome(s)	Proportion of Class I guideline indicated therapies received and occurrence of composite major adverse cardiac event. (See Table 3, main publication)
Key Secondary Outcomes	Health-related Quality of Life, duration of initial hospitalisation, unscheduled revascularisations, components of composite endpoint. (See Table 3, main publication)
