# Supplementary material for: Evaluation of the impact of the GRACE risk score on the management and outcome of patients hospitalised with non-ST elevation acute coronary syndrome in the UK: protocol of the UKGRIS cluster-randomised registry-based trial
Source: BMJ Open. 2019 Sep 5;9(9):e032165. doi: 10.1136/bmjopen-2019-032165 (PMC6731819; doi:10.1136/bmjopen-2019-032165)
Supplement: Supplementary data [file bmjopen-2019-032165supp002.pdf]

ONLINE SUPPLEMENTARY MATERIAL

Supplementary file 2 - Detailed derivation of primary outcome measure

Title

Evaluation of the impact of the GRACE risk score on the management and outcome of patients hospitalised with non-ST elevation acute coronary syndrome in the UK: protocol of the UKGRIS cluster-randomised registry-based trial.

Colin C. Everett, Keith A. A. Fox, Catherine Reynolds, Catherine Fernandez, Linda D. Sharples, Deborah D. Stocken, Kathryn Carruthers, Harry Hemingway, Andrew T. Yan, Shaun G. Goodman, David Brieger, Derek P. Chew, Chris P. Gale.

Supplementary File 2: Derivation of co-primary outcome measure: proportion of class I guideline care processes received

The overall definition is "Percentage of guideline processes followed correctly" (ie "appropriate care"). The numerator of the proportion will be the number of the below care processes received for which the patient was eligible, plus the number of care processes not received for which the patient was not eligible. The denominator of the proportion will be all guidelines in the following table, multiplied by the number of patients.

That is

$$\Pr(Guideline\ Process\ received) = \frac{\sum_{j,k} GP[Eligible\ and\ received]_{j,k} + \sum_{j,k} GP[Ineligible\ and\ not\ received]_{j,k}}{11 \times K}$$

Where $GP[-]_{j,k}$ takes the value 1 if the condition in the brackets is true (for guideline process j and patient k), and 0 if false. In the denominator, K represents the total number of participants. A summary for a single participant is a fraction out of 11.

Guideline process	Eligible if	Received if
1 – Aspirin	NSTEACS final diagnosis;<br>CRUSADE bleeding risk low or medium	Received during admission OR prescribed on discharge
2 – Ischaemia Testing	NSTEACS final diagnosis;<br>No angiography;<br>GRACE risk category = Low	Underwent one or more of Cardiac MRI, Radionuclide, Stress Echo or ETT during admission OR one more of these planned post discharge.
3 – Aspirin with P2Y12 inhibition	NSTEACS final diagnosis;<br>GRACE risk category = Intermediate / High	Received during admission OR prescribed on discharge
4 – Low molecular weight or unfractionated heparin	NSTEACS final diagnosis;<br>GRACE risk category = Intermediate / High	Received Low Molecular Weight heparin OR Unfractionated

		heparin OR fondaparinux during admission
5 – Coronary Angiography	NSTEACS final diagnosis; Angiography not inappropriate; GRACE risk category = Intermediate	Underwent angiography within 72 hours of admission, with or without PCI
6 – Urgent coronary angiography	NSTEACS final diagnosis; Angiography not inappropriate; GRACE risk category = High	Underwent angiography within 24 hours of admission, with or without PCI
7 – Left ventricular function assessment	NSTEACS final diagnosis;	Underwent one or more of Echo, Cardiac MRI, Radionuclide, Stress Echo during admission
8 – Angiotensin Converting Enzyme inhibitor / Angiotensin II receptor blocker	NSTEACS final diagnosis; one or more of the following:<br>• Diabetes<br>• Hypertension<br>• Congestive Cardiac Failure	Received one or more during admission OR prescribed on discharge
9 – Beta Blockers*	NSTEACS final diagnosis	Received during admission OR prescribed on discharge
10 – Statins	NSTEACS final diagnosis	Received during admission OR prescribed on discharge
11 – Cardiac rehabilitation	NSTEACS final diagnosis	Underwent during admission.

Table B1: Components of co-primary outcome measure, including criteria for eligibility and receipt.

Footnote * A sensitivity analysis will exclude the use of beta blockers from the set of guideline recommended care processes, and so the denominator of the proportion will be 10xK, rather than 11xK.
