# Supplementary material for: Evaluation of the impact of the GRACE risk score on the management and outcome of patients hospitalised with non-ST elevation acute coronary syndrome in the UK: protocol of the UKGRIS cluster-randomised registry-based trial
Source: BMJ Open. 2019 Sep 5;9(9):e032165. doi: 10.1136/bmjopen-2019-032165 (PMC6731819; doi:10.1136/bmjopen-2019-032165)
Supplement: Supplementary data [file bmjopen-2019-032165supp003.pdf]

ONLINE SUPPLEMENTARY MATERIAL

Supplementary file 3 - Detailed sample size calculation

Title

Evaluation of the impact of the GRACE risk score on the management and outcome of patients hospitalised with non-ST elevation acute coronary syndrome in the UK: protocol of the UKGRIS cluster-randomised registry-based trial.

Colin C. Everett, Keith A. A. Fox, Catherine Reynolds, Catherine Fernandez, Linda D. Sharples, Deborah D. Stocken, Kathryn Carruthers, Harry Hemingway, Andrew T. Yan, Shaun G. Goodman, David Brieger, Derek P. Chew, Chris P. Gale.

Supplementary file 3: Detailed Sample Size Calculation

1. For the co-primary endpoint of the proportion of class I guideline recommended therapies prescribed – to be analysed as a difference in proportions approach – we make the following assumptions:

- prescription rates in the Standard Care arm will be $\boldsymbol{\pi_0} = \mathbf{95}\%$,
- prescription rates in the GRACE arm will be $\boldsymbol{\pi_1} = \mathbf{98}\%$,
- 80% power
- 2-sided 5% significance test
- coefficient of variation of cluster event rates of $\boldsymbol{k} = \mathbf{0.02}$ and
- cluster size of $\boldsymbol{m} = \mathbf{100}$.

Based on these assumptions and the methods in Section 7.2.3 of [33], the number of clusters per arm is calculated using,

$$\boldsymbol{n} \geq \mathbf{1} + \frac{\left(\boldsymbol{z}_{\frac{\alpha}{2}} + \boldsymbol{z}_{\beta}\right)^2 \left(\left(\frac{\boldsymbol{\pi_0}(\mathbf{1} - \boldsymbol{\pi_0})}{\boldsymbol{m}}\right) + \left(\frac{\boldsymbol{\pi_1}(\mathbf{1} - \boldsymbol{\pi_1})}{\boldsymbol{m}}\right) + \boldsymbol{k}^2\left(\boldsymbol{\pi_0^2} + \boldsymbol{\pi_1^2}\right)\right)}{(\boldsymbol{\pi_0} - \boldsymbol{\pi_1})^2}$$

This results in a minimum of 14 clusters per arm (i.e. 2800 patients).

2. For the co-primary endpoint of time to composite endpoint occurrence – to be analysed as a time-to-event outcome – we make the following assumptions:

- exponential incidence rate in the Standard Care arm of $\boldsymbol{\lambda_0} = \mathbf{13}\%$ per year,
- exponential incidence rate in the Grace arm of $\boldsymbol{\lambda_1} = \mathbf{10.4}\%$ per year (20% reduction),
- mean follow-up of 27 months (based on 30 months recruitment period and 12 months follow up),

- 80% power
- 2-sided 5% significance test
- coefficient of variation of cluster event rates of $\boldsymbol{k} = \mathbf{0.05}$ and
- cluster size of $\boldsymbol{m} = \mathbf{100}$.

Then using the exponential distribution analogue (Given in Section 7.2.1 of [33]) of the above equation,

$$\boldsymbol{n} \geq \mathbf{1} + \frac{\left(\boldsymbol{z}_{\frac{\alpha}{2}} + \boldsymbol{z}_{\beta}\right)^{2}\left(\boldsymbol{\lambda}_{0} + \boldsymbol{\lambda}_{1} + \boldsymbol{k}^{2}\left(\boldsymbol{\lambda}_{0}^{2} + \boldsymbol{\lambda}_{1}^{2}\right)\right)}{(\boldsymbol{\lambda}_{0} - \boldsymbol{\lambda}_{1})^{2}}$$

we require a minimum of 14 clusters per arm (2800 patients).

To allow for attrition of 7-10%, a minimum of 15 clusters of size 100, per arm, will be recruited to ensure adequate power.
