# Supplementary material for: Evaluation of the impact of the GRACE risk score on the management and outcome of patients hospitalised with non-ST elevation acute coronary syndrome in the UK: protocol of the UKGRIS cluster-randomised registry-based trial
Source: BMJ Open. 2019 Sep 5;9(9):e032165. doi: 10.1136/bmjopen-2019-032165 (PMC6731819; doi:10.1136/bmjopen-2019-032165)
Supplement: Supplementary data [file bmjopen-2019-032165supp004.pdf]

ONLINE SUPPLEMENTARY MATERIAL

Supplementary file 4 – UKGRIS Trial Management Structure

Title

Evaluation of the impact of the GRACE risk score on the management and outcome of patients hospitalised with non-ST elevation acute coronary syndrome in the UK: protocol of the UKGRIS cluster-randomised registry-based trial.

Colin C. Everett, Keith A. A. Fox, Catherine Reynolds, Catherine Fernandez, Linda D. Sharples, Deborah D. Stocken, Kathryn Carruthers, Harry Hemingway, Andrew T. Yan, Shaun G. Goodman, David Brieger, Derek P. Chew, Chris P. Gale.

Supplementary File 4: UKGRIS Trial Management Structure

Trial Management Group: Prof. C. P. Gale (Chief Investigator), Dr C. Fernandez (Project Delivery Lead), Ms C. Reynolds (Trial and Data Manager), Mr C. C. Everett (statistician), Prof L. D. Sharples (Project Methodology Lead), Prof. H. Hemingway (epidemiologist), Prof. K. A. A. Fox (cardiologist), Prof D. D. Stocken (supervising statistician and study scientific lead), Mrs K. Carruthers (Nurse specialist).

Data Monitoring and Ethics Committee: Prof R. Storey (cardiologist), Dr P. Ludman (cardiologist), Dr A McConnachie (statistician), Dr M. de Belder (Chair, cardiologist).

Trial Steering Committee: Prof. A. Timmis (Chair, cardiologist), Prof. M. Mamas (cardiologist), Mr G. Oliver (patient representative), Dr L Gray (statistician).
