# Supplementary material for: Evaluation of the impact of the GRACE risk score on the management and outcome of patients hospitalised with non-ST elevation acute coronary syndrome in the UK: protocol of the UKGRIS cluster-randomised registry-based trial
Source: BMJ Open. 2019 Sep 5;9(9):e032165. doi: 10.1136/bmjopen-2019-032165 (PMC6731819; doi:10.1136/bmjopen-2019-032165)
Supplement: Reviewer comments [file bmjopen-2019-032165.reviewer_comments.pdf]

PEER REVIEW HISTORY

BMJ Open publishes all reviews undertaken for accepted manuscripts. Reviewers are asked to complete a checklist review form (**http://bmjopen.bmj.com/site/about/resources/checklist.pdf**) and are provided with free text boxes to elaborate on their assessment. These free text comments are reproduced below.

ARTICLE DETAILS

**TITLE (PROVISIONAL)**	Evaluation of the impact of the GRACE risk score on the management and outcome of patients hospitalised with non-ST elevation acute coronary syndrome in the UK: protocol of the UKGRIS cluster-randomised registry-based trial.
**AUTHORS**	Everett, Colin; Fox, Keith; Reynolds, Catherine; Fernandez, Catherine; Sharples, Linda; Stocken, Deborah; Carruthers, Kathryn; Hemingway, Harry; Yan, Andrew; Goodman, Shaun; Brieger, D; Chew, Derek; Gale, Chris

This article was not externally reviewed at BMJ Open. Protocols that have been independently assessed prior to submission to BMJ Open are usually fast-tracked to publication on the grounds that further substantial changes will not be possible. This independent assessment will usually be external, independent review for both a substantial grant award from a non-commercial or government funder and ethics approval.
